# Anabolic-Androgenic Steroids and Cardiometabolic Derangements

**DOI:** 10.7759/cureus.12492

**Published:** 2021-01-05

**Authors:** Khashayar Farzam

**Affiliations:** 1 Family Medicine, University of Iowa Hospitals and Clinics, Iowa City, USA

**Keywords:** metabolism, family medicine, internal medicine, lipidology, anabolic androgenic steroid, cardiology, lipid metabolism, anabolic steroid, men’s health, testosterone hormone

## Abstract

Anabolic-androgenic steroids (AAS) are commonly used among both competitive athletes and recreational athletes in order to gain a performance edge. Unfortunately, AAS generally carries a broad range of short term and long term adverse effects. These include endocrinological abnormalities, cardiovascular risks, psychological issues, and largely adverse effects on every organ system in the human body. While testosterone, and at times oxandrolone, are used for clinical purposes, AAS generally encompasses a very broad range of synthetic compounds that are used at high doses. In this case report, we look at a patient who has used the vast majority of common anabolic steroids over the past three decades and how these compounds may affect long-term metabolic and cardiovascular health. The purpose is to provide a primary care approach to this patient population.

## Introduction

Anabolic-androgenic steroids (AAS) are synthetic hormones that enhance human performance. Through their inception in the 1930s, their breadth has evolved and both recreational users and athletes readily utilize these agents for various purposes [1]. AAS is largely divided into intramuscular injectable and oral forms. They are traditionally used in cycles ranging from several weeks to several months, though this has evolved in recent years to longitudinal use. Classically, an injectable testosterone ester is used as a base compound with several other injectable and oral compounds used in a cycle. While testosterone, and rarely other compounds, carry medical purposes, most compounds largely exist for performance enhancement reasons.

Anabolic steroids carry various side effects both in the short term and long term. Superficial side effects include androgenic alopecia and acne. Other immediate health effects can include hypertension and dyslipidemia. Orally consumed anabolic steroids are predominantly hepatotoxic, especially at higher doses that are used for performance enhancement. Some highly popular injectable steroids pose the risk of nephrotoxicity. These compounds also cause hormonal dysfunction, especially hypogonadism. Some of these adverse effects are seen from testosterone use, but many others are specific to the agents being used [2]. There are also unpredictable long-term effects of certain anabolic agents including metabolic derangements and cardiotoxicity [3].

Testosterone is readily available for prescription use and most physicians who care for the appropriate patient population are able to oversee its usage [2]. There is drastically increased difficulty for physicians when dealing with other anabolic steroids since nearly all of them are not sanctioned for medical purposes or even human use. However, there is a large growing population who has used or continues to use these agents. This creates the need for a greater understanding of these drugs, especially in the context of screening for adverse health effects. During the present time, there is no formal evidence-based guidance on screening anabolic steroid users for various pathologies. Physicians are likely to rely on national screening guidelines due to the lack of evidence on primary prevention in this patient population. Increased recognition of AAS and improved knowledge of this patient population is important, particularly given that over 1 million Americans have used anabolic steroids [4].

## Case presentation

The patient is a 56-year-old Caucasian male who presented to the family medicine clinic for a general check-up for health maintenance. He has a past medical history of obstructive sleep apnea (OSA), pituitary adenoma which was successfully treated with cabergoline, and extensive anabolic steroid use. He had no immediate medical or social concerns and he also denied any exertional chest pain or dyspnea. He was not on any prescription medications but did endorse recreational use of testosterone cypionate at 125 mg per week. Supplement use was significant for garlic tablets, tauroursodeoxycholic acid (TUDCA) at 500 mg daily, resveratrol, alpha-lipoic acid (ALA) at 600 mg daily, turmeric, vitamin C, fish oil at 3 grams daily, vitamin D at 5000 IU daily, coenzyme Q10, and n-acetyl cysteine (NAC) 1200 mg daily. He did not smoke tobacco and only drank alcohol socially. The patient had a blood pressure of 136/80. Body mass index was 35.6 kg/m2. Vital signs were otherwise normal. He had no abnormal physical exam findings and was in a low body fat percentage range on visual examination.

His anabolic steroid history was quite significant. He first began using anabolic steroids over thirty years ago and has used these agents in cycles up until the present day. This included a broad range of injectable and oral anabolic steroids. He had used various esters of injectable testosterone consistently since starting this regime. Other examples of anabolic steroids used included trenbolone, nandrolone decanoate, methenolone, stanozolol (Winstrol), boldenone undecylenate (Equipoise), oxymetholone (Anadrol), metandienone (Dianabol), and fluoxymesterone (Halotesin). These compounds were used sporadically in various cycles over the years. 

Lab workup was obtained which included a complete blood count (CBC), creatinine, electrolyte panel, hemoglobin A1C, total prostate-specific antigen (PSA), lipid panel, and a basic liver function panel. The patient’s hemoglobin was elevated at 18.0 g/dl but his CBC was otherwise unremarkable. Creatinine was elevated at 1.4 mg/dl, likely due to high levels of muscle mass. Cystatin C levels were within normal limits which made kidney disease unlikely. Electrolyte panel along with liver enzymes and other liver function tests were normal, as was the patient’s PSA. Surprisingly, the patient’s hemoglobin A1C was elevated at 6.4%. Upon further inquiry, he had a prior hemoglobin A1C of 6.9% in the recent past which was never followed upon. His result was fully diagnostic for diabetes mellitus. What was surprising about this result, was the diagnosis of diabetes mellitus despite his low-body fat levels and very high levels of physical activity throughout his life. Lipid panel returned significant for low-density lipoprotein (LDL) level of 168 mg/dl, high-density lipoprotein (HDL) level of 52 mg/dl, and triglyceride level of 78 mg/dl.

Urine studies obtained included a urinalysis and urine protein: creatinine ratio, both of which were normal. More specialized tests were done to further risk stratify this patient and included apolipoprotein A-1 (159 mg/dl), apolipoprotein B100 (137 mg/dl) and high-sensitivity C-reactive protein levels (hs-CRP) (1.5 mg/dl).

**Figure 1:**
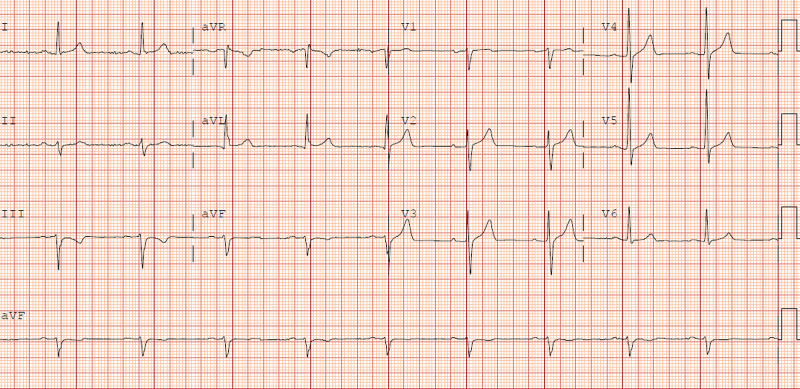
Electrocardiogram (ECG) Sinus rhythm with findings suggestive of left axis deviation.

Electrocardiogram was obtained and was significant for left axis deviation but otherwise no other significant findings (Figure 1). An echocardiogram was done on a subsequent visit and revealed mild left ventricular hypertrophy and mildly decreased right ventricular function. The patient’s ejection fraction (EF) was 50%-55%. Of important note, he never had any significant hypertension documented throughout his life. These echocardiogram findings were suggestive of direct AAS induced biventricular effects.

On further follow up, there was a further inquiry made in regards to his hemoglobin A1C and his unexpected diagnosis of diabetes mellitus. The patient had a vague and possible family history of type 1 diabetes mellitus and had previously been evaluated for this at a younger age but never had a formal diagnosis made. This prompted an evaluation for possible latent autoimmune diabetes in adults (LADA). Fasting C-peptide levels were obtained and were 1.7 ng/ml. Insulin antibodies, Islet cell antibodies, and glutamic acid decarboxylase antibody (GAD) were obtained and negative. An autoimmune component was ruled out and his overall diagnosis was consistent with type 2 diabetes mellitus (T2DM).

The combination of this patient’s T2DM diagnosis, hyperlipidemia, borderline elevated blood pressure, and moderately elevated hs-CRP was consistent with metabolic derangements that are generally not seen in athletes who maintain lower body fat levels and engage in rigorous exercise. His echocardiogram findings were also abnormal for an athlete without chronic hypertension. The patient was placed on aspirin 81 mg daily. He was not amenable to statin therapy and opted for major lifestyle modification and niacin therapy for his dyslipidemia, though his LDL goal given his T2DM diagnosis would be <70 mg/dl. He was also started on an angiotensin-converting enzyme (ACE) inhibitor at a low dose to aid in his left ventricular remodeling and better optimize his hypertension.

This case highlights the potential need for extensive metabolic and cardiac evaluation in anabolic steroid users due to various unknown risks associated with long term AAS use. While there is some reasonable understanding of the effects of testosterone use alone, most anabolic steroids that are very commonly used are poorly studied and hence not well understood.

## Discussion

AAS use and exogenous supplementation of testosterone are very similar yet distinctly different. Exogenous testosterone is unlikely to cause direct pathologic cardiac effects and has been associated with some improved metabolic biomarkers [5]. On the contrary, other anabolic steroids carry unknown metabolic risks. Worsened insulin sensitivity as a result of AAS usage has been previously described in the literature [6], but there is limited patient data to support it. A major limitation in assessing this patient population are numerous confounding variables such as supplemental use of insulin for weight gain purposes and excess body fat levels. The latter is most prominent among heavyweight powerlifters and strongman competitors. In contrast, athletes who maintain lower body fat levels generally do not develop major insulin resistance, especially in the absence of an autoimmune component [7].

Current screening guidelines by the United States Preventative Services Task Force (USPSTF) recommend screening for dyslipidemia in men starting at the age of 35, unless they have an identifiable risk factor for coronary artery disease [8]. In addition, it is recommended to screen for abnormal blood glucose levels in those who are obese or have risk factors for diabetes, also starting at age 40 [8]. Given the general association of athletes and optimal metabolic health, it is possible that physicians may minimize screening this patient population. Furthermore, cardiac screening in athletes with an electrocardiogram or echocardiogram is generally not advised. Yet there is consistent evidence that AAS can directly induce direct pathologic changes to the heart [9]. Specific knowledge regarding AAS can help physicians more accurately screen this patient population, especially given many of the unknown long term side effects.

## Conclusions

Anabolic steroids carry many potential and somewhat unknown long term risks, such as metabolic derangements and cardiac toxicity. Greater physician knowledge could be helpful in accurately screening these patients prior to serious complications. More research is needed to provide better insight on addressing this patient population. 

## References

[1] Hoberman JM, Yesalis CE (1995). The history of synthetic testosterone. Sci Am.

[2] Hartgens F, Kuipers H (2004). Effects of androgenic-anabolic steroids in athletes. Sports Med.

[3] Karila TAM, Karjalainen JE, Mäntysaari MJ, Viitasalo MT, Seppälä TA (2003). Anabolic androgenic steroids produce dose-dependent increase in left ventricular mass in power athletes, and this effect is potentiated by concomitant use of growth hormone. Int J Sports Med.

[4] Pope HG Jr, Wood RI, Rogol A, Nyberg F, Bowers L, Bhasin S (2014). Adverse health consequences of performance-enhancing drugs: an endocrine society scientific statement. Endocr Rev.

[5] Traish AM, Saad F, Guay A (2009). The dark side of testosterone deficiency: II. Type 2 diabetes and insulin resistance. J Androl.

[6] Livingstone C, Collison M (2002). Sex steroids and insulin resistance. Clin Sci (Lond).

[7] Tarp J, Støle AP, Blond K, Grøntved A (2019). Cardiorespiratory fitness, muscular strength and risk of type 2 diabetes: a systematic review and meta-analysis. Diabetologia.

[8] (2020). United States Preventive Services Taskforce: recommendation: abnormal blood glucose and type 2 diabetes mellitus: screening. https://www.uspreventiveservicestaskforce.org/uspstf/recommendation/screening-for-abnormal-blood-glucose-and-type-2-diabetes.

[9] Torrisi M, Pennisi G, Russo I (2020). Sudden cardiac death in anabolic-androgenic steroid users: a literature review. Medicina.

